# Microscopic and submicroscopic *Plasmodium falciparum* infection, maternal anaemia and adverse pregnancy outcomes in Papua New Guinea: a cohort study

**DOI:** 10.1186/s12936-019-2931-7

**Published:** 2019-09-02

**Authors:** Holger W. Unger, Anna Rosanas-Urgell, Leanne J. Robinson, Maria Ome-Kaius, Shadrach Jally, Alexandra J. Umbers, Willie Pomat, Ivo Mueller, Eline Kattenberg, Stephen J. Rogerson

**Affiliations:** 1 Centre for Maternal and Newborn Health, Liverpool School of Tropical Medicine, Liverpool, UK; 2 Institute of Tropical Medicine, Antwerp, Belgium; 3 Vector Borne Diseases Unit, PNG Institute of Medical Research, Goroka, Papua New Guinea; 4 Burnet Institute, Melbourne, Australia; 5 Walter and Eliza Hall Institute of Medical Research, Parkville, Australia; 6 Department of Medical Biology, University of Melbourne, Parkville, Australia; 7 Institut Pasteur, Paris, France; 8 Department of Medicine, (RMH), Peter Doherty Institute for Infection and Immunity, The University of Melbourne, 792 Elizabeth Street, Melbourne, VIC 3000 Australia

**Keywords:** Anaemia, Diagnosis, Fetal growth retardation, Low birth weight, Malaria, *Plasmodium falciparum*, Pregnancy outcome, Premature birth

## Abstract

**Background:**

Infection during pregnancy with *Plasmodium falciparum* is associated with maternal anaemia and adverse birth outcomes including low birth weight (LBW). Studies using polymerase chain reaction (PCR) techniques indicate that at least half of all infections in maternal venous blood are missed by light microscopy or rapid diagnostic tests. The impact of these subpatent infections on maternal and birth outcomes remains unclear.

**Methods:**

In a cohort of women co-enrolled in a clinical trial of intermittent treatment with sulfadoxine–pyrimethamine (SP) plus azithromycin for the prevention of LBW (< 2500 g) in Papua New Guinea (PNG), *P. falciparum* infection status at antenatal enrolment and delivery was assessed by routine light microscopy and real-time quantitative PCR. The impact of infection status at enrolment and delivery on adverse birth outcomes and maternal haemoglobin at delivery was assessed using logistic and linear regression models adjusting for potential confounders. Together with insecticide-treated bed nets, women had received up to 3 monthly intermittent preventive treatments with SP plus azithromycin or a single clearance treatment with SP plus chloroquine.

**Results:**

A total of 9.8% (214/2190) of women had *P. falciparum* (mono-infection or mixed infection with *Plasmodium vivax*) detected in venous blood at antenatal enrolment at 14–26 weeks’ gestation. 4.7% of women had microscopic, and 5.1% submicroscopic *P. falciparum* infection. At delivery (n = 1936), 1.5% and 2.0% of women had submicroscopic and microscopic *P. falciparum* detected in peripheral blood, respectively. Submicroscopic *P. falciparum* infections at enrolment or at delivery in peripheral or placental blood were not associated with maternal anaemia or adverse birth outcomes such as LBW. Microscopic *P. falciparum* infection at antenatal enrolment was associated with anaemia at delivery (adjusted odds ratio [aOR] 2.00, 95% confidence interval [CI] 1.09, 3.67; P = 0.025). Peripheral microscopic *P. falciparum* infection at delivery was associated with LBW (aOR 2.75, 95% CI 1.27; 5.94, P = 0.010) and preterm birth (aOR 6.58, 95% CI 2.46, 17.62; P < 0.001).

**Conclusions:**

A substantial proportion of *P. falciparum* infections in pregnant women in PNG were submicroscopic. Microscopic, but not submicroscopic, infections were associated with adverse outcomes in women receiving malaria preventive treatment and insecticide-treated bed nets. Current malaria prevention policies that combine insecticide-treated bed nets, intermittent preventive treatment and prompt treatment of symptomatic infections appear to be appropriate for the management of malaria in pregnancy in settings like PNG.

## Background

Infection with the malaria parasite *Plasmodium falciparum* during pregnancy is detrimental to both mother and the developing fetus. It causes maternal anaemia and can lead to significant maternal morbidity and death, in particular in low-transmission settings [1]. Sequestration of *P. falciparum*-infected erythrocytes in the placental intervillous space disturbs transplacental nutrient transport and creates a reservoir of inflammation [2]. As a consequence, infected mothers are also more likely to deliver low birth weight babies (LBW; < 2500 g) [1, 3]. Therefore, *P. falciparum* infection is a principal cause of LBW and infant death in endemic settings [3]. LBW, which can be due to preterm birth (PTB) and/or fetal growth restriction, has significant short and long-term negative impacts [4].

Whilst light microscopy (LM) and/or rapid diagnostic tests are used in clinical practice to detect infection in women self-presenting to health facilities with symptoms, research studies using sensitive polymerase chain reaction (PCR) techniques have helped to unravel the true burden of infection in pregnancy. Many of these studies suggest that at least half of *P. falciparum* infections in peripheral maternal blood are missed by LM [5–10]. Submicroscopic *P. falciparum* infections were associated with maternal anaemia [5, 9, 10] and LBW [5, 8, 11, 12] in some but not all studies [6, 7, 13]. The impact of submicroscopic placental *P. falciparum* infections is less well described but limited evidence suggests these are associated with LBW [12]. Mixed *P. falciparum*/*Plasmodium vivax* submicroscopic infections may be associated with PTB, but submicroscopic *P. vivax* mono-infections were not associated with anaemia or LBW [13, 14]. Monthly intermittent preventive treatment of malaria in pregnancy with sulfadoxine–pyrimethamine (SP), a strategy implemented to treat occult placental infection, clears submicroscopic *P. falciparum* infections but does not prevent re-infection in the interval between treatments [6].

The present study evaluates the associations between microscopic and submicroscopic *P. falciparum* infections at antenatal enrolment and at delivery and maternal haemoglobin at delivery or adverse pregnancy outcomes in a cohort of pregnant Papua New Guinean (PNG) women. Participants were co-enrolled in a clinical trial evaluating intermittent preventive treatment with SP plus azithromycin (SPAZ) for the prevention of LBW. Adverse pregnancy outcomes assessed included LBW, PTB (< 37 weeks of gestation) and measuring small-for-gestational age at birth as a proxy for fetal growth restriction.

## Methods

### Study design and setting

A prospective cohort study of pregnant women was undertaken from November 2009 until February 2013 at nine antenatal clinics and health centres in Madang Province on the North Coast of PNG. Women’s malaria infection status was assessed by quantitative real-time PCR and LM at study inclusion, and participants were followed up for birth outcomes including miscarriage, stillbirth, LBW, PTB and fetal growth restriction, and maternal haemoglobin was measured at delivery. Maternal malaria infection status was assessed at delivery using peripheral and placental blood. A subset of women had microscopy and PCR examination of peripheral blood at their second and third antenatal study visits (1 and 2 months following the enrolment visit, respectively) to assess point prevalence.

The study setting has been described in detail previously [15]. In brief, there is perennial transmission of both *P. falciparum* and *P. vivax* and a high burden of adverse pregnancy outcomes, particularly LBW (17%) and maternal anaemia (90% of pregnant women had a haemoglobin < 110 g/L) [8]. Women in the cohort study were co-enrolled in a clinical trial assessing the impact of 3 monthly intermittent preventive treatments with SPAZ on LBW [15]. Whenever possible, women were provided with insecticide-treated bed nets and given oral iron and folic acid supplementation, as per national policy. The estimated antenatal HIV prevalence in the region was 1%. Women not randomized to SPAZ received the control treatment (single malaria clearance treatment at study inclusion with SP plus chloroquine) as per current national policy. Multiple pregnancies and women with an estimated gestational age greater than 26 gestational weeks by symphysis-fundal height were excluded. The parent trial demonstrated that SPAZ significantly reduced the risks of LBW and PTB [15].

### Clinical assessments

Birth weights were measured using digital infant scales (Cupid 1, Charder Medical, Taiwan; accuracy 10 g). Pregnancy losses before an estimated 22 gestational weeks were categorized as miscarriages. Haemoglobin levels were estimated using by HemoCue (Angelholm, Sweden; accuracy of 1 g/L). Anaemia was defined as a haemoglobin < 110 g/L. A sub-set of women in the study underwent ultrasound dating of their pregnancy (Logiqbook XP, General Electric Medical Systems, UK), as described previously [15]. Birth before 37 gestational weeks was defined as PTB. Small-for-gestational age (SGA) was defined as a birth weight below the 10th centile of the Intergrowth-21 standard [16].

### Laboratory analysis

Maternal peripheral blood smears were prepared from venous blood samples taken at enrolment and at delivery. Air-dried blood smears were stained for 30 min with 4% Giemsa. The number of asexual parasites per 200 leukocytes (or per 500 if < 10 parasites/200 leukocytes) were counted on thick blood smear, assuming 8000 leukocytes/μL of blood. Smears were judged negative following examination of 200 oil-immersion fields without detection of parasites. Each slide was assessed by two microscopists, and discrepant results were resolved by third reads, or when necessary, by qPCR [17]. qPCR was used to detect *P. falciparum* and *P. vivax* infections in maternal venous blood samples collected in EDTA anti-coagulant. DNA was extracted from 200 μL whole blood with QIAamp96 DNA Blood Mini Kit (QIAGEN, Valencia, CA) and eluted to a final volume of 200 μL dH20, and analysed as previously described (qPCR sensitivity: 1 parasite per μL) [18].

For each infection assessment *P. falciparum* infection status was coded ‘negative’ if both LM and qPCR were negative for *P. falciparum*, ‘submicroscopic infection’ if the thick smear was negative but qPCR detected *P. falciparum*, and ‘microscopic infection’ if both thick smear and qPCR detected *P. falciparum*. Women with incomplete data for LM and qPCR at study inclusion, and women with positive LM but negative qPCR (i.e. possible *Plasmodium ovale*/*Plasmodium malariae* infections or false positive smears), were excluded from the study.

### Statistical analysis

Linear regression models were designed to assess the association between *P. falciparum* infection status, classified as submicroscopic infection versus no infection or as microscopic infection versus no infection, and continuous parametric outcome measures (birth weight, haemoglobin). Logistic regression models were constructed to assess the association between *P. falciparum* infection status and bivariate outcome measures (pregnancy loss, LBW, PTB, SGA and anaemia).

All singleton pregnancies with complete LM/qPCR data at study inclusion that were successfully followed up for birth outcome were considered in the pregnancy loss analysis. Birth weight analyses were confined to singleton congenitally normal live born babies for whom a birth weight measurement was available.

Analyses of pregnancy outcomes were adjusted a priori for factors previously identified as determinants of birth weight in the cohort, namely treatment arm of the parent clinical trial, sex of the baby, gravidity, number of study visits, enrolment clinic, bed net use, maternal nutritional status, stunting, and socio-economic status, and timing of birth weight measurement (not included as covariate in PTB analyses) [15]. Haemoglobin analyses were adjusted a priori for treatment arm, gravidity, betel nut consumption, number of study visits, enrolment clinic, bed net use, nutritional status, stunting, socio-economic status and timing of haemoglobin measurement [19].

*Plasmodium falciparum* infections included mono-infections and dual infections with *P. vivax*. *Plasmodium vivax* mono-infections were not associated with any of the adverse outcomes under scrutiny in this study or in a published multi-centre observational study to which part of the present cohort contributed [14], and analyses were thus not adjusted for their presence.

Analyses were conducted separately for infection status at enrolment (prospective cohort study) and infection status at delivery (cross-sectional analysis). In view of the fact that only half of women had infection status assessments at second and third study visits, and given comparatively the low infection prevalence at these time points, these data not included in outcome analyses but are presented to describe infection prevalence between the first antenatal visit and delivery. In addition, associations were assessed between infection status at both enrolment and delivery combined, and analyses were performed stratified on gravidity. A P-value < 0.05 was considered statistically significant. Findings are presented in line with guidance for the reporting of observational studies.

### Ethical considerations

The study was approved by the PNG Institute of Medical Research (PNGIMR) Institutional Review Board (0815), the PNG Medical Research Advisory Council (08.01), and the Melbourne Health Human Research Ethics Committee (2008.162). The study was conducted in accordance with Good Clinical Practice guidelines (ICH GCP E6).

## Results

**Fig. 1:**
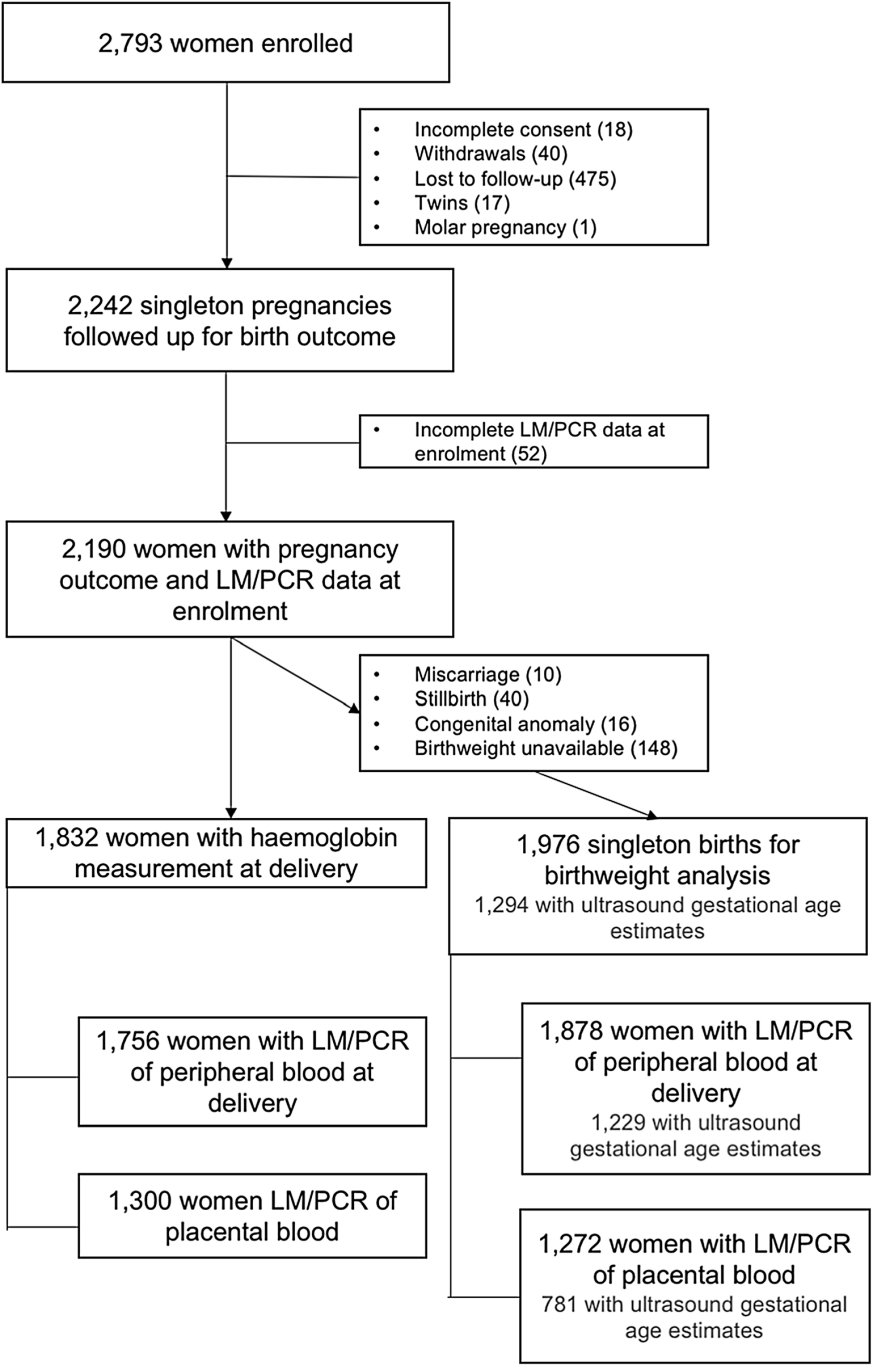
Study flowchart. *LM* light microscopy, *PCR* polymerase-chain reaction

Of 2793 women enrolled in the parent clinical trial, 2190 had singleton pregnancies and complete LM/qPCR data at inclusion and were successfully followed up for birth outcome. Amongst them, 1976 babies were suitable for inclusion in the birth weight analysis, and 1832 women had their haemoglobin levels measured at birth (Fig. 1).

The mean gestational age at enrolment by symphysis-fundal height was 21.1 weeks (standard deviation [SD], 4.2 weeks), 49.0% of women were primigravid, and 50.3% were randomized to SPAZ.

A total of 9.8% (n = 214) of women had *P. falciparum* detected in venous blood at enrolment and (n = 61) of women had *P. vivax* mono-infections (Table 1). *Plasmodium falciparum* infection prevalence at interim antenatal visits, assessed in a subset of women only, was low (Table 1). At delivery 3.5% of women had *P. falciparum* infection detected in peripheral venous blood, and 4.4% in placental blood (Table 1). Only 11 women (0.6%) had *P. falciparum* infections detected in peripheral blood at both enrolment and delivery.

**Table 1:** Point prevalence malaria infection at study visits

Study visit	No infection % (n)	Submicroscopic infection % (n)	Microscopic infection % (n)
Enrolment (n = 2190)			
*P. falciparum*^a^	90.2 (1976)	5.1 (111)	4.7 (103)
*P. vivax* mono-infection	97.2 (2129)	2.2 (48)	0.6 (13)
Second study visit (n = 1015)			
*P. falciparum*	98.5 (1000)	0.7 (7)	0.8 (8)
Third study visit (n = 859)			
*P. falciparum*	97.8 (841)	1.1 (9)	1.1 (9)
Delivery—peripheral blood (n = 1936)			
*P. falciparum*	96.5 (1869)	1.5 (29)	2.0 (38)
*P. vivax* mono-infection	98.3 (1904)	1.6 (30)	0.1 (2)
Delivery—placental blood (n = 1300)			
*P. falciparum*^b^	96.6 (1256)	1.1 (14)	2.3 (30)
*P. vivax* mono-infection	99.5 (1293)	0.5 (7)	0.0 (0)

^a^ *P. falciparum* mono-infection or mixed infection with *P. vivax* (n = 12)

^b^ *P. falciparum* mono-infection or mixed infection with *P. vivax* (n = 1)

There were 40 stillbirths and 10 miscarriages. Amongst women with a congenitally normal live birth and who were followed up for birth weight (n = 1976), 15.2% (n = 301) of babies were LBW and mean birth weight was 2943 g (SD 477). Ultrasound dating was undertaken for 1294 (65.5%) of these pregnancies: 9.3% and 24.7% of babies were born preterm and SGA, respectively. The mean haemoglobin at delivery was 100.5 g/L (SD 17). Three quarters of women (74.2%, n = 1360) were anaemic at delivery.

### *Plasmodium falciparum* infection at first antenatal visit and adverse pregnancy outcomes

Submicroscopic and microscopic *P. falciparum* infections were not associated with stillbirth or miscarriage, LBW, PTB and SGA (Table 2). The adjusted mean birth weight difference was − 2 g (95% confidence interval [CI] − 94, 90; P = 0.97) for submicroscopic *P. falciparum* infection, and − 12 g (95% CI − 108, 83; P = 0.80) for microscopic infection.

**Table 2:** Associations between *Plasmodium falciparum* infection at antenatal enrolment and miscarriage/stillbirth, low birthweight, preterm birth and fetal growth restriction

Infection status	% (N/N total)	Adjusted odds ratio	95% confidence interval	*P*
Miscarriage/stillbirth^a^ (n = 2190)				
No infection	2.2 (44/1976)			
Submicroscopic infection	1.8 (2/111)	0.81	(0.19, 3.41)	0.77
Microscopic infection	3.9 (4/103)	1.96	(0.68, 5.66)	0.22
Low birthweight^b^ (n = 1976)				
No infection	15.1 (270/1786)			
Submicroscopic infection	14.3 (14/98)	1.01	(0.55, 1.84)	0.98
Microscopic infection	18.5 (17/92)	0.97	(0.54, 1.75)	0.93
Preterm birth^c^ (n = 1294)				
No infection	8.9 (105/1181)			
Submicroscopic infection	1.7 (1/60)	0.17	(0.02, 1.26)	0.08
Microscopic infection	11.3 (6/53)	1.22	(0.49, 1.26)	0.67
Small for gestational age^b^ (n = 1294)				
No infection	24.5 (289/1181)			
Submicroscopic infection	33.3 (20/60)	1.65	(0.93, 2.92)	0.09
Microscopic infection	20.8 (11/53)	0.71	(0.35, 1.43)	0.34

^a^ Adjusted for treatment arm, gravidity, enrolment clinic, bed net use, undernutrition, stunting, ethnicity and socio-economic status

^b^ Adjusted for treatment arm, gender of the baby, gravidity, number of study visits, enrolment clinic, bed net use, nutritional status, stunting, socio-economic status and timing of birthweight measurement

^c^ Adjusted for treatment arm, gender of the baby, gravidity, number of study visits, enrolment clinic, bed net use, nutritional status, stunting, and socio-economic status

### Peripheral and placental *P. falciparum* infection at delivery and adverse pregnancy outcomes

Microscopic but not submicroscopic *P. falciparum* infections at delivery were associated with LBW and PTB in the cohort (Table 3). The association between microscopic infection at enrolment and LBW was more pronounced amongst primigravid (aOR 3.49; 95% CI 1.35, 9.04) compared to multigravid women (aOR 2.31, 95% CI 0.75, 7.14). The adjusted mean birth weight difference was − 48 g (95% CI − 214, 118; P = 0.57) for submicroscopic, and − 126 g (95% CI − 273, 20.8; P = 0.09) for microscopic *P. falciparum* infection.

**Table 3:** Association of microscopic and submicroscopic *Plasmodium falciparum* infection at delivery in maternal peripheral venous blood and placental blood with low birthweight, preterm birth and fetal growth restriction

Infection status	% (N/N total)	Adjusted odds ratio	95% confidence interval	*P*
*P. falciparum* infection at delivery (peripheral blood)				
Low birthweight^a^ (n = 1878)				
No infection	15.0 (272/1812)			
Submicroscopic infection	17.2 (5/29)	1.00	(0.35, 2.83)	0.99
Microscopic infection	29.7 (11/37)	2.75	(1.27, 5.94)	0.010
Preterm birth^b^ (n = 1229)				
No infection	8.2 (97/1190)			
Submicroscopic infection	5.9 (1/17)	0.54	(0.07, 4.29)	0.56
Microscopic infection	31.8 (7/22)	6.58	(2.46, 17.62)	< 0.001
Small for gestational age^a^ (n = 1229)				
No infection	24.1 (287/1190)			
Submicroscopic infection	29.4 (5/17)	1.32	(0.44, 3.96)	0.63
Microscopic infection	31.8 (7/22)	1.71	(0.67, 4.39)	0.26
*P. falciparum* infection at delivery (placental blood)				
Low birthweight^a^ (n = 1272)				
No infection	13.8 (170/1228)			
Submicroscopic infection	28.6 (4/14)	2.85	(0.82, 9.91)	0.10
Microscopic infection	30.0 (9/30)	2.08	(0.87, 4.98)	0.10
Preterm birth^b^ (n = 781)				
No infection	6.9 (52/749)			
Submicroscopic infection	18.2 (2/11)	3.34	(0.66, 16.83)	0.15
Microscopic infection	19.1 (4/21)	3.04	(0.89, 10.3)	0.08
Small for gestational age^a^ (n = 781)				
No infection	21.4 (160/749)			
Submicroscopic infection	36.4 (4/11)	2.60	(0.71, 9.56)	0.15
Microscopic infection	33.3 (7/21)	1.62	(0.61, 4.34)	0.34

^a^ Adjusted for treatment arm, gender of the baby, gravidity, number of study visits, enrolment clinic, bed net use, nutritional status, stunting, socio-economic status and timing of birthweight measurement

^b^ Adjusted for treatment arm, gender of the baby, gravidity, number of study visits, enrolment clinic, bed net use, nutritional status, stunting, and socio-economic status

The adjusted mean birth weight difference for placental submicroscopic infection was − 179 g (95% CI − 417, 60; P = 0.14), and for placental microscopic infection − 154 g (95% CI − 320, 11; P = 0.07). Neither microscopic nor submicroscopic placental *P. falciparum* infection was significantly associated with LBW, PTB or SGA (Table 3).

### *Plasmodium falciparum* infection and anaemia at delivery

Women with microscopic but not submicroscopic *P. falciparum* infection at enrolment were more likely to be anaemic at delivery compared to women with no infection (Table 4). The association between microscopic infection at enrolment and anaemia at delivery was more pronounced amongst multigravid (aOR 2.41; 95% CI 1.12, 5.17) compared to primigravid women (aOR 1.92, 95% CI 0.74, 5.00). The adjusted mean difference in maternal haemoglobin at delivery by infection status at enrolment was -0.7 g/L (95% CI − 4.3, 2.9; P = 0.69) for submicroscopic *P. falciparum* infection, and − 2.6 (95% CI − 6.1, 1.1; P = 0.17) for microscopic *P. falciparum* infection.

**Table 4:** Association of *Plasmodium falciparum* infection at antenatal enrolment and at delivery and anaemia (haemoglobin < 110 g/L) at delivery

Infection status	% (N/N total)	Adjusted odds ratio^a^	95% confidence interval	*P*
*P. falciparum* infection at enrolment (n = 1832)				
No infection	73.9 (1218/1649)			
Submicroscopic infection	69.2 (63/91)	0.78	(0.49, 1.24)	0.29
Microscopic infection	85.9 (79/92)	2.00	(1.09, 3.67)	0.025
*P. falciparum* infection at delivery (peripheral blood) (n = 1756)				
No infection	74.2 (1261/1700)			
Submicroscopic infection	79.2 (19/24)	1.15	(0.42, 3.16)	0.79
Microscopic infection	78.1 (25/32)	1.15	(0.49, 2.72)	0.75
*P. falciparum* infection at delivery (placental blood) (n = 1174)				
No infection	71.7 (815/1137)			
Submicroscopic infection	71.4 (10/14)	0.87	(0.27, 2.81)	0.81
Microscopic infection	78.3 (18/23)	1.28	(0.47, 3.53)	0.63

^a^ Adjusted for treatment arm, gravidity, betel nut consumption, number of study visits, enrolment clinic, bed net use, nutritional status, stunting, socio-economic status and timing of haemoglobin measurement

For parasitaemia at delivery, the adjusted mean difference for maternal haemoglobin at delivery was − 2.5 g/L (95% CI − 9.4, 4.3; P = 0.47) for submicroscopic *P. falciparum* infection, and − 5.9 g/L (95% CI − 11.8, 0.1; P = 0.05) for microscopic infection. There was no statistically significant increase in the odds of anaemia in women with microscopic and submicroscopic *P. falciparum* infection at delivery compared to women with no infection (Table 4).

For placental malaria, the adjusted mean difference for maternal haemoglobin at delivery was − 1.1 g/L (95% CI − 10.1, 7.8; P = 0.80) for submicroscopic *P. falciparum* infection, and − 6.2 g/L (95% CI − 13.2, 7.8; P = 0.09) for microscopic infection. Placental *P. falciparum* infection was not significantly associated with maternal anaemia at delivery (Table 4).

### Association of infection at enrolment with infection at delivery

There was no association between *P. falciparum* infection (submicroscopic or microscopic) at antenatal enrolment with peripheral blood infection at delivery (P = 0.42). Microscopic infection at enrolment (P < 0.001), but not submicroscopic (P = 0.38) infection was associated with *P. falciparum* infection in placental blood. When peripheral *P. falciparum* infection at enrolment and delivery were combined, microscopic infection was associated with preterm birth (aOR 2.54, CI 1.30–4.97, P = 0.006, Additional file 1)

## Discussion

In this cohort of PNG women receiving at least one dose of SP-IPTp submicroscopic *P. falciparum* infections detected at first antenatal visit (~ 14–26 weeks of gestation) or at delivery were not associated with maternal anaemia or adverse birth outcomes such as LBW. Peripheral microscopic *P. falciparum* infection at antenatal enrolment was associated with anaemia at delivery, and at delivery it was associated with LBW and PTB. LM underestimated infection prevalence at first antenatal visit by more than 50%.

The relationship between submicroscopic *P. falciparum* and pregnancy outcomes remains contentious, and the present study is not the only one to report an apparent lack of association between submicroscopic peripheral infection and adverse maternal [7, 13] and infant outcomes at birth [6, 13, 20]. Studies demonstrating no association are characterized by a comparatively low infection burden, suggesting lack of power may be an issue. Other factors, such as differences in malaria prevention approaches, ethnic differences, proportion of primigravidae in the sample, transmission intensity, and the type of PCR assay used, may play a role. In women not receiving or defaulting from IPTp the burden of submicroscopic infections will be higher and such infections are likely to develop into higher density and chronic infections, both of which are associated with adverse outcomes. Individual participant data meta-analysis may reveal the true impact of submicroscopic infections and could account for some of the foregoing factors. In contrast to another study [12], placental microscopic and submicroscopic infection were not associated with adverse outcomes, although there were trends towards a deleterious effect for both infection categories.

Neither microscopic or submicroscopic *P. falciparum* infection detected at study inclusion were associated with adverse birth outcomes, suggesting anti-malarial treatments given as part of the clinical trial cleared most infections or reduced parasite densities and limited deleterious downstream effects. Microscopic infections detected in peripheral blood at delivery were associated with adverse birth outcomes, re-affirming the deleterious impact of microscopic *P. falciparum* infection for both mother and fetus. Such late infections may represent recrudescence, or new infections acquired in later pregnancy. Indeed, the proportion of women infected with *P. falciparum* fell following study enrolment and first treatment, remaining low at second and third treatment visits, but resurged at delivery, as reported from another longitudinal study [5]. Many study participants did not receive anti-malarials in the last 2 months of pregnancy, and this finding supports the policy of continuing monthly IPTp until delivery.

Microscopic but not submicroscopic infection was associated with anaemia at delivery. Akin to birth outcomes there is heterogeneity in the reported impact of submicroscopic *P. falciparum* on maternal anaemia. The apparent lack of association between submicroscopic *P. falciparum* and anaemia in this study may be due to relatively low infection prevalence and sample size, and potential confounders such as iron and folic acid supplementation, prescribed to all study participants, may be of importance.

*Plasmodium vivax* mono-infections, most of which were submicroscopic, were not associated with any of the adverse outcomes assessed in this cohort, reflecting findings from a recent multi-centre study which included a subset of women from this cohort in their analyses [14]. Research from Colombia found an increased risk of PTB amongst women with submicroscopic mixed *P. falciparum*/*P. vivax* infection, but not *P. vivax* or *P. falciparum* submicroscopic mono-infections [13]. The small number of mixed infections in the present cohort precluded a meaningful assessment of their impact and they were consequently grouped together with *P. falciparum* mono-infections in the analyses.

The strengths of this study include its large sample size, longitudinal design and assessment of infection at enrolment and in both peripheral and placental blood at delivery. Important limitations include the lack of parasite genotyping data to distinguish recrudescent from novel infections, the lack of obstetric ultrasound for a third of pregnancies, and the comparatively low infection burden, potentially affecting power of the study to detect associations between infection and adverse outcomes. The sample size of the present study was based on power calculations in relation to the impact of the trial intervention on outcomes rather than infection status, and infection burden was lower than anticipated at trial design. The authors recognize that this may be one of the reasons explaining the apparent lack of association of submicroscopic infections with adverse outcomes, and wide confidence intervals reflect the uncertainty of risk estimates. Lastly, women received bed nets, malaria preventive treatment and close follow-up under trial conditions, thereby limiting generalizability of study findings to women receiving routine or no antenatal care.

## Conclusions

Microscopy misses at least half of peripheral blood infections. Submicroscopic infections are common in pregnant women, and are hard to diagnose, raising concerns about their potential to affect maternal and fetal health. In the present study, there was limited evidence that these infections are associated with adverse outcomes in the context of peripheral infection burden of < 10% and provision of bed nets and preventive treatment. Until more sensitive diagnostic tools are available, insecticide-treated bed nets, monthly preventive treatment until delivery and detection and treatment of symptomatic infections appear appropriate for the management of malaria in pregnancy in settings like PNG.

## Supplementary information

**Additional file 1: Table S1.** Association between peripheral *Plasmodium falciparum* infection status during pregnancy (enrolment and delivery combined) and low birthweight, preterm birth, small-for-gestational age at birth, and anaemia (haemoglobin < 110 g/L) at delivery.

## Data Availability

Data are available from the WWARN data repository (http://www.wwarn.org/working-together/sharing-data/accessing-data) for researchers who meet the criteria for access to confidential data, and from the corresponding author on reasonable request.

## Abbreviations

aOR: adjusted odds ratio
CI: confidence interval
DNA: deoxyribonucleic acid
IPTp: intermittent preventive treatment in pregnancy
LBW: low birth weight
LM: light microscopy
PNG: Papua New Guinea
PTB: preterm birth
qPCR: quantitative real-time polymerase chain reaction
SD: standard deviation
SGA: small for gestational age
SP: sulfadoxine–pyrimethamine
SPAZ: sulfadoxine–pyrimethamine plus azithromycin

## References

[1] Rogerson SJ, Desai M, Mayor A, Sicuri E, Taylor SM, van Eijk AM (2018). Burden, pathology, and costs of malaria in pregnancy: new developments for an old problem. Lancet Infect Dis.

[2] Rogerson SJ, Hviid L, Duffy PE, Leke RF, Taylor DW (2007). Malaria in pregnancy: pathogenesis and immunity. Lancet Infect Dis.

[3] Walker PG, Ter Kuile FO, Garske T, Menendez C, Ghani AC (2014). Estimated risk of placental infection and low birthweight attributable to *Plasmodium falciparum* malaria in Africa in 2010: a modelling study. Lancet Glob Health.

[4] McCormick MC (1985). The contribution of low birth weight to infant mortality and childhood morbidity. N Engl J Med.

[5] Cottrell G, Moussiliou A, Luty AJ, Cot M, Fievet N, Massougbodji A (2015). Submicroscopic *Plasmodium falciparum* infections are associated with maternal anemia, premature births, and low birth weight. Clin Infect Dis.

[6] Cohee LM, Kalilani-Phiri L, Boudova S, Joshi S, Mukadam R, Seydel KB (2014). Submicroscopic malaria infection during pregnancy and the impact of intermittent preventive treatment. Malar J.

[7] Elbadry MA, Tagliamonte MS, Raccurt CP, Lemoine JF, Existe A, Boncy J (2017). Submicroscopic malaria infections in pregnant women from six departments in Haiti. Trop Med Int Health.

[8] Stanisic DI, Moore KA, Baiwog F, Ura A, Clapham C, King CL (2015). Risk factors for malaria and adverse birth outcomes in a prospective cohort of pregnant women resident in a high malaria transmission area of Papua New Guinea. Trans R Soc Trop Med Hyg.

[9] Mayor A, Moro L, Aguilar R, Bardaji A, Cistero P, Serra-Casas E (2012). How hidden can malaria be in pregnant women? Diagnosis by microscopy, placental histology, polymerase chain reaction and detection of histidine-rich protein 2 in plasma. Clin Infect Dis.

[10] Mockenhaupt FP, Rong B, Till H, Eggelte TA, Beck S, Gyasi-Sarpong C (2000). Submicroscopic *Plasmodium falciparum* infections in pregnancy in Ghana. Trop Med Int Health.

[11] Adegnika AA, Verweij JJ, Agnandji ST, Chai SK, Breitling LP, Ramharter M (2006). Microscopic and sub-microscopic *Plasmodium falciparum* infection, but not inflammation caused by infection, is associated with low birth weight. Am J Trop Med Hyg.

[12] Mohammed AH, Salih MM, Elhassan EM, Mohmmed AA, Elzaki SE, El-Sayed BB (2013). Submicroscopic *Plasmodium falciparum* malaria and low birth weight in an area of unstable malaria transmission in Central Sudan. Malar J.

[13] Gavina K, Gnidehou S, Arango E, Hamel-Martineau C, Mitran C, Agudelo O (2018). Clinical outcomes of submicroscopic infections and correlates of protection of VAR2CSA antibodies in a longitudinal study of pregnant women in Colombia. Infect Immun.

[14] Bardaji A, Martinez-Espinosa FE, Arevalo-Herrera M, Padilla N, Kochar S, Ome-Kaius M (2017). Burden and impact of *Plasmodium vivax* in pregnancy: a multi-centre prospective observational study. PLoS Negl Trop Dis.

[15] Unger HW, Ome-Kaius M, Wangnapi RA, Umbers AJ, Hanieh S, Suen CS (2015). Sulphadoxine–pyrimethamine plus azithromycin for the prevention of low birthweight in Papua New Guinea: a randomised controlled trial. BMC Med.

[16] Villar J, Cheikh Ismail L, Victora CG, Ohuma EO, Bertino E, Altman DG (2014). International standards for newborn weight, length, and head circumference by gestational age and sex: the Newborn Cross-Sectional Study of the INTERGROWTH-21st Project. Lancet.

[17] Umbers AJ, Unger HW, Rosanas-Urgell A, Wangnapi RA, Kattenberg JH, Jally S (2015). Accuracy of an HRP-2/panLDH rapid diagnostic test to detect peripheral and placental *Plasmodium falciparum* infection in Papua New Guinean women with anaemia or suspected malaria. Malar J.

[18] Rosanas-Urgell A, Mueller D, Betuela I, Barnadas C, Iga J, Zimmerman PA (2010). Comparison of diagnostic methods for the detection and quantification of the four sympatric *Plasmodium* species in field samples from Papua New Guinea. Malar J.

[19] Ome-Kaius M, Unger HW, Singirok D, Wangnapi RA, Hanieh S, Umbers AJ (2015). Determining effects of areca (betel) nut chewing in a prospective cohort of pregnant women in Madang Province, Papua New Guinea. BMC Pregnancy Childbirth.

[20] Mankhambo L, Kanjala M, Rudman S, Lema VM, Rogerson SJ (2002). Evaluation of the OptiMAL rapid antigen test and species-specific PCR to detect placental *Plasmodium falciparum* infection at delivery. J Clin Microbiol.

