# Supplementary material for: Microscopic and submicroscopic Plasmodium falciparum infection, maternal anaemia and adverse pregnancy outcomes in Papua New Guinea: a cohort study
Source: Malar J. 2019 Sep 2;18:302. doi: 10.1186/s12936-019-2931-7 (PMC6720091; doi:10.1186/s12936-019-2931-7)
Supplement: Supplementary file 1 — Additional file 1: Table S1. Association between peripheral Plasmodium falciparum infection status during pregnancy (enrolment and delivery combined) and low birthweight, preterm birth, small-for-gestational age at birth, and anaemia (haemoglobin < 110 g/L) at delivery. [file 12936_2019_2931_MOESM1_ESM.docx]

**Table S1** Association between peripheral *Plasmodium falciparum* infection status during pregnancy (enrolment and delivery combined) and low birthweight, preterm birth, small-for-gestational age at birth, and anaemia (haemoglobin < 110 g/L) at delivery

| **Infection status** | **% (N/N total)** | **Adjusted**  **odds ratio** | **95% confidence interval** | ***P*** |
| --- | --- | --- | --- | --- |
| *Low birthweight*^1^ (n=1,878) | | | | |
| No infection | 14.9 (244/1,641) |  |  |  |
| Submicroscopic infection | 16.4 (19/116) | 1.12 | (0.66, 1.92) | 0.67 |
| Microscopic infection | 20.7 (25/121) | 1.27 | (0.77, 2.08) | 0.34 |
|  |  |  |  |  |
| *Preterm birth*^2^ (n=1,229) | | | | |
| No infection | 8.3 (90/1,086) |  |  |  |
| Submicroscopic infection | 2.8 (2/72) | 0.29 | (0.07, 1.22) | 0.09 |
| Microscopic infection | 18.3 (13/71) | 2.54 | (1.30, 4.97) | 0.006 |
|  |  |  |  |  |
| *Small for Gestational Age*^1^ (n=1,229) | | | | |
| No infection | 23.9 (259/1,086) |  |  |  |
| Submicroscopic infection | 33.3 (24/72) | 1.70 | (1.00, 2.89) | 0.05 |
| Microscopic infection | 22.5 (16/71) | 0.89 | (0.49, 1.62) | 0.71 |
|  |  |  |  |  |
| *Anaemia at delivery* (1,756) | | | | |
| No infection | 73.9 (1,132/1,532) |  |  |  |
| Submicroscopic infection | 70.4 (76/108) | 0.80 | (0.51, 1.23) | 0.30 |
| Microscopic infection | 83.6 (97/116) | 1.67 | (1.00, 2.80) | 0.05 |

^1^ adjusted for treatment arm, gender of the baby, gravidity, number of study visits, enrolment clinic, bed net use, nutritional status, stunting, socio-economic status and timing of birthweight measurement.

^2^ adjusted for treatment arm, gender of the baby, gravidity, number of study visits, enrolment clinic, bed net use, nutritional status, stunting, and socio-economic status.
